# Prevalence of avoidant/restrictive food intake disorder in children and adolescents with rare diseases

**DOI:** 10.1186/s13023-026-04233-5

**Published:** 2026-01-31

**Authors:** Johannes Boettcher, Thomas Lücke, Holger Zapf, Anne Daubmann, Jonas Denecke, Mathilde Kersting, Hermann Kalhoff, Skadi Beblo, Ricarda Schmidt, Alena Thiele, Sarah Hohmann, Wieland Kiess, Ania C. Muntau, Anja Hilbert, Silke Wiegand-Grefe

**Affiliations:** 1 Department of Child and Adolescent Psychiatry, Psychotherapy and Psychosomatics, University Medical Center Hamburg-Eppendorf, Martinistrasse 52, 20246 Hamburg, Germany; 2 Research Department of Child Nutrition, University Hospital of Pediatrics and Adolescent Medicine, Ruhr University Bochum, Bochum, Germany; 3 Department of Medical Biometry and Epidemiology, University Medical Center Hamburg–Eppendorf, Hamburg, Germany; 4 Department of Pediatrics, University Medical Center Hamburg-Eppendorf, Hamburg, Germany; 5 German Center for Child and Adolescent Health (DZKJ), Partner Site Hamburg, University Medical Center Hamburg-Eppendorf, 20246 Hamburg, Germany; 6 Pediatric Clinic Dortmund, Dortmund, Germany; 7 Department of Women and Child Health, Hospital for Children and Adolescents, Center for Pediatric Research Leipzig (CPL), University Hospital, University of Leipzig, Leipzig, Germany; 8 Department of Psychosomatic Medicine and Psychotherapy, Integrated Research and Treatment Center AdiposityDiseases, Behavioral Medicine Research Unit, University of Leipzig Medical Center, Leipzig, Germany; 9 German Center for Child and Adolescent Health (DZKJ), Partner Site Leipzig/Dresden, University of Leipzig Medical Center, Leipzig, Germany; 10 Department of Psychiatry and Psychotherapy, University Medical Center Hamburg-Eppendorf, Hamburg, Germany

**Keywords:** Rare diseases, ARFID, Selective eating, Food avoidance

## Abstract

**Background:**

Children and adolescents with rare diseases represent a population that may be at risk for an avoidant/restrictive food intake disorder (ARFID). This study aimed to quantitatively examine the symptoms of ARFID in a sample of children and adolescents with rare diseases and their association with sociodemographic characteristics, eating disorder psychopathology, health-related quality of life (HRQoL), and mental health.

**Methods:**

In this observational study, data from 309 families of children with rare diseases drawn from a multicenter clinical study were used to estimate the prevalence of ARFID symptoms in children and adolescents aged 8–21 years in Germany was estimated via self-report (*n* = 169) and parent report (*n* = 502). Differences between the sample of individuals with rare diseases and normative population data, between those with and without ARFID symptoms, and between subgroups of rare diseases were investigated. Additionally, correlations between eating disorder psychopathology, sociodemographic, and psychosocial variables were investigated.

**Results:**

The prevalence of symptoms of ARFID was relatively high, with a relevant difference between self-report (3.6%) and parent report (10.2%). A vast proportion of self-reported ARFID symptoms were relevantly higher in children and adolescents with rare diseases than in normative population data. Moreover, the presence of ARFID symptoms in both self-report and parent report versions suggests that these symptoms may be consistent across varying severity levels of coexisting rare diseases. Both self-reported and parent reported ARFID symptoms in children and adolescents with rare diseases were associated with impaired HRQoL and mental health.

**Conclusion:**

The study underscores the importance of considering ARFID in children and adolescents with rare diseases. The comorbidity of rare diseases and ARFID may impact clinical care; however, healthcare professionals often lack sufficient knowledge of these patient populations. Therefore, further research and consideration in clinical practice are necessary.

**Trial registration:**

German Clinical Trials Register: DRKS00015859 (registered 18 December 2018) and ClinicalTrials.gov: NCT04339465 (registered 8 April 2020).

**Supplementary Information:**

The online version contains supplementary material available at 10.1186/s13023-026-04233-5.

## Background

Avoidant/restrictive food intake disorder (ARFID) is a heterogeneous eating disorder applied to individuals consuming a significantly low amount and/or variety in food intake, leading to severe physical and/or psychosocial impairment, which is not driven by body image concerns, but rather by a lack of interest in eating, sensory sensitivities to food, or a fear of aversive consequences such as choking or vomiting [1, 2]. ARFID typically emerges in childhood and is accompanied by mental and somatic comorbidities, with early adverse events—such as congenital diseases and prematurity—suggested as potential, though not definitively established, risk factors requiring further investigation [3, 4]. Previous research on ARFID has mostly focused on pediatric samples, including specialized treatment service samples, documenting high psychiatric comorbidities with anxiety disorders and neurodevelopmental disorders like autism spectrum disorder [5–9]. However, research on ARFID and the specific challenges faced by children and adolescents with rare diseases, such as health-related quality of life (HRQoL) and mental health issues, has been neglected so far.

Rare diseases are characterized by a low prevalence varying by region [10] and are defined in the European Union as having a prevalence of less than 1:2000 [11]. Despite the heterogeneity in disease patterns, rare diseases often share common characteristics, such as severe and chronic progression, limited life expectancy, early onset in childhood, and other symptoms [12]. Some rare diseases can lead to avoidance of food due to pain, discomfort, or other digestive issues [13–15], whereas others may have symptoms that affect sensory processing, leading to a heightened sensitivity to tastes, textures, and smells [16], which can contribute to the development of ARFID. Although evidence on environmental and genetic risk factors for ARFID is limited, some rare diseases may contribute to its development [17–19]. Strikingly, the specific challenges associated with ARFID can have a detrimental effect on the HRQoL and mental health of children and adolescents with rare diseases, as shown in a cohort of adolescents and young adults with cystic fibrosis [15].

Given the complex interplay between medical complications, nutritional challenges, and psychological impacts inherent in rare diseases, an examination of symptoms of ARFID in children and adolescents with a rare disease has the potential to offer novel insights and perspectives on the understanding of both conditions [18]. Therefore, this study sought to investigate [1] the prevalence of symptoms of ARFID in children and adolescents with rare diseases; [2] to compare self-reported ARFID symptoms with normative population data; [3] to compare the prevalence of self-reported and parent reported ARFID symptoms; [4] to compare symptoms of ARFID, HRQoL and mental health in children and adolescents with and without ARFID symptoms; and [5] to examine the associations between ARFID symptoms, HRQoL, and mental health. We hypothesized that the symptoms of ARFID would be more prevalent in children and adolescents with rare diseases compared to normative data. We also hypothesized discrepancies between self-reported and parent reported ARFID symptoms, with parents likely reporting more severe symptoms than children and adolescents themselves. Furthermore, we hypothesized that children and adolescents with ARFID symptoms would exhibit lower HRQoL and lower mental health compared to their counterparts without ARFID symptoms. Finally, we hypothesized that ARFID symptoms would show negative associations with HRQoL and mental health within this population.

## Methods

### Participants and procedures

The informants included families of children and adolescents with rare diseases in the rater-blinded, randomized, controlled, multicenter trial *Children Affected by Rare Disease and Their Network (CARE-FAM-NET)* [20]. The recruitment was conducted at 17 sites, which were located all over Germany. Baseline data on participants were investigated with standardized psychometric questionnaires. Parents provided written informed consent, and adolescents aged ten years or older also provided informed assent before participation. Participants were able to withdraw from the study at any time. The baseline measurement took place from January 2019 to February 2021. The study received ethical approval from the Ethics Committee of the Medical Chamber Hamburg (PV 5749). Additionally, ethical approval was obtained from the responsible boards at all study sites. The study was preregistered at the German Clinical Trials Register: DRKS00015859 (registered December 18, 2018) and ClinicalTrials.gov: NCT04339465 (registered April 8, 2020).

Of the 687 families included in the CARE-FAM-NET trial, 309 (45.0%) families, solely those with an eight-year-old or older child, were included to ensure the validated age range for the questionnaires. We extended the inclusion to adolescents up to age 21, aligning with current broader definitions that consider physical maturation and delayed social transitions [21], as well as the availability of treatment options for children and adolescents up to age 21 in child and adolescent psychiatry and psychotherapy in Germany. Of these families, one family (0.3%) with three affected children, 15 families (4.9%) with two affected children, and 293 families (94.8%) with one affected child participated. Parent report data were available from 502 parents, including 107 mothers (21.3%), nine fathers (1.8%), and 193 mother-father dyads (38.4% mothers and fathers). Self-report data were available from 169/309 (54.7%) children and adolescents aged ten years and over.

### Measures

#### Sociodemographic and clinical characteristics

Sociodemographic characteristics such as age, gender, and number of children, as well as parental age, education, and employment status, were collected. All participating families had at least one child who met the criteria for a rare disease as defined by the European Commission [11], and each diagnosis was confirmed by medical personnel prior to inclusion. Furthermore, the various rare diseases were assigned to superordinate groups based on the Orphanet classification system [22]. To account for the severity of the disease, in addition to the common categories, three superordinate groups adapted from Noeker (2013) [23] were used, including diseases with an episodic-relapsing course (e.g., epilepsy), diseases with permanent functional limitations and disability (e.g., chromosomal defects and congenital malformations), and diseases with a progressive or life-threatening course (e.g., neuromuscular diseases, oncological diseases). The main sociodemographic and clinical characteristics are shown in Table 1.

**Table 1:** Sociodemographic and clinical characteristics of children and adolescents with rare diseases and their parents

	Self-report data (*n* = 169)		Parent report data (*n* = 502)	
**Characteristics**	M	SD	M	SD
Patient’s age (years)	14.4	2.85	12.6	3.31
Mothers age (years)	45.3	6.58	44.0	6.90
Fathers age (years)	57.3	4.73	47.6	7.08
Number of children in the family	2.15	0.97	2.2	0.93
Number of children with rare diseases	1.1	0.40	1.1	0.41
**Children**	*n*	%	*n*	%
Gender				
Male	86	52.8	257	51.2
Female	77	47.8	245	48.8
**Parents**	*n*	%	*n*	%
Gender				
Male	-	-	300	59.8
Female	-	-	202	40.2
Marital status				
Single	-	-	36	7.2
With life partner	-	-	26	5.2
Married	-	-	366	72.9
Divorced	-	-	39	7.8
Widowed	-	-	5	1.0
Highest school qualification				
Primary school	-	-	54	10.8
Secondary school	-	-	170	33.9
High school diploma	-	-	217	43.2
Without qualification	-	-	1	0.2
Other qualification	-	-	11	2.2
Highest professional qualification				
Apprenticeship	-	-	203	40.4
Master craftsman training	-	-	53	10.6
Technical college/ University	-	-	137	27.3
Without qualification	-	-	7	1.4
Other qualification	-	-	25	5.0
**Clinical variables**	*n*	%	*n*	%
Rare disease groups				
Cardiac congenital and functional diseases	1	0.6	7	1.4
Chromosomal defects and congenital malformations	9	5.5	42	8.4
Chronic inflammatory disease	16	9.8	35	7.0
Chronic intestinal diseases	1	0.6	2	0.4
Disorders of the central nervous system	3	1.8	16	3.2
Endocrinological diseases (congenital/acquired)	3	1.8	8	1.6
Epilepsy (with or without genetics)	5	3.1	33	6.6
Hematological diseases	2	1.2	7	1.4
Kidney diseases and diseases of the urinary tract	9	5.5	15	3.0
Metabolic diseases	23	17.8	96	19.1
Neurocutaneous diseases	9	5.5	37	7.4
Neuromuscular diseases	39	23.9	100	19.9
Oncological diseases	10	6.1	16	3.2
Other syndromes and symptom complexes without genetics	4	2.5	18	3.6
Other syndromes with genetic causes	18	11.0	57	11.4
Pulmonary and respiratory diseases	5	3.1	13	2.6
Disease groups according to Noeker (2013)				
Diseases with an episodic-relapsing course	25	14.8	67	13.3
Diseases with permanent functional limitations & disability	85	50.3	289	57.6
Diseases with a progressive or life-threatening course	57	33.7	146	29.1

Note. *M =* Mean, *SD =* Standard deviation. ^1^Higher education is defined as having at least a high school or a higher degree

#### Symptoms of ARFID

The Eating Disorders in Youth-Questionnaire (EDY-Q) [24] was used as a measure of symptoms of ARFID based on self-report and parent report. The EDY-Q comprises 14 items, which are answered on a seven-point rating scale ranging from 0 = never to 6 = always, and cover three ARFID variants, food avoidance emotional disorder (FAED), selective eating (SE), and functional dysphagia (FD), as well as pica and rumination disorder (not reported here) [25]. The total mean score was used to measure the overall symptoms of ARFID; higher scores indicated a greater presence of ARFID symptoms. Furthermore, to document the presence of symptoms of ARFID, the diagnostic coding described in previous research was used for both the self-report and parent report versions. In addition, to document the presence of symptoms of ARFID, the EDY-Q items *lack of interest in food* or *avoidance to try new foods* or *sensory food avoidance* with [26] or without [27] the *underweight* item was required to be reported at least often (≥ 4), while the items *weight concern* and *shape concern* had to be reported less than sometimes (< 3) following the two previously proposed EDY-Q categories. Moreover, the diagnostic coding for broad symptoms of ARFID proposed by Schmidt et al., (2018) was used, including the additional items *food avoidance* or *emotional food avoidance* or *selective eating behavior* or *avoidance to try new food* or *fear of choking* reported at least often. Lastly, the current data were compared to recent normative population data for ages 7 to14 years (*M* = 10.5, *SD* = 2.0) [28]. The German self-report version of the EDY-Q has shown sufficient psychometric properties [27, 29, 30]. In this study the observed internal consistency of the total score was questionable for self-reported (α = 0.61) and acceptable for parent reported (α = 0.74) scores of the EDY-Q. Interrater agreement between self-reported and parent reported data of the EDY-Q was poor to moderate (Intraclass Correlation (ICC) = 0.532), with a 95% confidence interval of 0.407 to 0.637.

#### Eating disorder psychopathology

The Eating Disorder Examination-Questionnaire 8 for Children (ChEDE-Q8) [31] was used to measure children’s eating disorder psychopathology based on self-report ≥ 8 years. The ChEDE-Q8 comprises eight items, which are answered on a seven-point rating scale, ranging from 0 = feature was absent to 6 = feature was present every day or to an extreme degree. The aggregated mean score was used as an indicator of global eating disorder psychopathology; higher scores indicated greater psychopathology. The German version of the ChEDE-Q8 has been shown to have good psychometric properties [31]. The observed internal consistency for the global score of the ChEDE-Q8 was excellent (α = 0.91).

#### Health-related quality of life

The DISABKIDS Chronic Generic Modul-37 (DCGM-37) [32] was used to assess self-reported and parent reported HRQoL in children and adolescents with rare diseases. The DCGM-37 comprises 37 items, which are answered on a five-point rating scale, ranging from 1 = never to 5 = always. The DCGM-37 includes the three main domains of mental, social, and physical HRQoL, as well as the six subscales. Additionally, a total HRQoL score can be calculated. In this study, the linearly transformed total score ranging from 0 to 100 was used, with higher scores indicating better HRQoL. The DCGM-37 has shown acceptable psychometric properties in children and adolescents with chronic diseases [33]. The observed internal consistency was good for both the self-reported (α = 0.80) and parent reported (α = 0.81) total scores of the DCGM-37. Interrater agreement between self-reported and parent reported data of the DCGM-37 was moderate to good (ICC = 0.736), with a 95% confidence interval of 0.656 to 0.799.

#### Mental health

The Youth Self-Report/11-18R and Child Behavior Checklist/6-18R (YSR/11-18R and CBCL/6-18R) [34] were used to assess mental health in self-report and parent report. The YSR/11-18R and the CBCL/6-18R consist of 112 and 113 items, which are answered on a three-point rating scale ranging from 0 = not true to 2 = very true or often true. The YSR/11-18R and the CBCL/6-18R include the three composites total score, internalizing problems, and externalizing problems, as well as eight symptom clusters. In this study, solely the total scores of the YSR/11-18R and CBCL/6-18R were used, with higher scores indicating greater impairment. The psychometric properties of YSR/11-18R and CBCL/6-18R have been shown to be acceptable [34]. The observed internal consistency was excellent for both the self-reported (α = 0.94) and parent reported (α = 0.93) total scores of the YSR/11-18R and CBCL/6-18R. Interrater agreement between self-reported and parent reported data of the YSR/11-18R and CBCL/6-18R was poor to moderate (ICC = 0.478), with a 95% confidence interval of 0.346 to 0.591.

### Data analytic plan

The data analysis was performed using descriptive statistics and bivariate tests. One-sample *t*-tests were used to examine differences between the rare diseased sample and normative population data. Dependent two-sample *t*-tests were used to investigate differences between maternal and paternal reports. Mann-Whitney *U* tests were used to examine differences between children and adolescents with and without symptoms of ARFID. Kruskal-Wallis *H* tests were used to examine differences between subgroups of rare diseases. Cohen’s *d*, Cramer’s *V*, Hedges *g* and $\:{\eta\:}^{2}$ were used as measures of effect size. Intraclass correlation coefficients (ICC) using a two-way random effects model were used to assess the level of agreement between self-reported and parent reported data. Based on the 95% confidence interval (CI) of ICC estimates, values were considered as poor (< 0.50), moderate (0.50 − 0.75), good (0.75 − 0.90), and excellent (> 0.90) agreement [35]. Pearson correlations were used to analyze associations between variables. Missing values were not imputed, and adjustments for multiple testing were not made. Statistical significance was set at *p* $\:<$ 0.05 (two-tailed). As these were post-hoc analyses, the *p*-values were considered descriptively. Statistical analyses were conducted using SPSS Statistics 29 and GraphPad Prism 9.

## Results

### Symptoms of ARFID

#### Prevalence

In the rare disease sample, the prevalence of symptoms of ARFID was 3.6% (*n* = 6 of 169) according to self-report and 10.2% (*n* = 51 of 502) according to parent report. The prevalence of broad symptoms of ARFID was 7.2% (*n* = 15) according to self-report and 11.6% (*n* = 58) according to parent report. The self-reported and parent reported prevalence differed across different EDY-Q diagnostic codings, with effect sizes ranging from small to moderate. See Table 2 for more information on the prevalence of symptoms of ARFID.

**Table 2:** Overview of EDY-Q categories previously reported in the literature for the self-report (*n* = 169) and parent report samples (*n* = 502)

EDY-Q diagnostic coding	EDY-Q items	Self-report	Parent report	Fisher’s exact test	
		*n* (%)	*n* (%)	*p*	V
Symptoms of ARFID without underweight problems^a^	(items 2 OR 10 OR 12 ≥ 4) AND (item 6 < 3 and item 7 < 3)	29/169 (17.2)	115/502 (22.9)	**0.034**	0.18
Symptoms of ARFID with underweight problems^b^	(items 2 OR 10 OR 12 ≥ 4) AND (item 4 ≥ 4) AND (item 6 < 3 and item 7 < 3)	6/169 (3.6)	51/502 (10.2)	**< 0.001**	0.32
Broad symptoms of ARFID with underweight problems^c^	(items 1 OR 2 OR 3 OR 8 OR 9 OR 10 OR 11 OR 12 ≥ 4) AND (item 4 ≥ 4) AND (item 6 < 3 and item 7 < 3)	15/154 (7.2)	58/502 (11.6)	**0.005**	0.27
Broad symptoms of ARFID without underweight problems^c^	(items 1 OR 2 OR 3 OR 8 OR 9 OR 10 OR 11 OR 12 ≥ 4) AND (item 6 < 3 and item 7 < 3)	59/110 (34.9)	177/502 (35.3)	**< 0.001**	0.31

Note. EDY-Q = Eating Disorders in Youth – Questionnaire. ARFID = Avoidant/Restrictive Food Intake Disorder. ^a^ Kurz, S., van Dyck, Z., Dremmel, D., Munsch, S., & Hilbert, A. (2015). Early-onset restrictive eating disturbances in primary school boys and girls. European Child & Adolescent Psychiatry, 24, 779–785. ^b^ van Dyck, Z. & Hilbert, A. (2016). Eating Disorders in Youth-Questionnaire. Deutsche Version. Universität Leipzig: http://nbn-resolving.de/urn:nbn:de:bsz:15-qucosa-197236. ^c^ Schmidt, R., Vogel, M., Hiemisch, A., Kiess, W., & Hilbert, A. (2018). Pathological and non-pathological variants of restrictive eating behaviors in middle childhood: A latent class analysis. *Appetite*, *127*, 257–265. Fisher’s exact test was solely conducted with cases that had both self-report and parent report data

#### Comparison of rare disease sample and normative population data

The prevalence of self-reported symptoms of ARFID with EDY-Q items reported at least ‘often’ ($\:\ge\:4$) did differ between the rare diseased sample and normative population data on the items *underweight*, *wish to gain weight*, *shape concern*, *selective eating behavior*, *avoidance to try new food*, and *sensory avoidance*, with prevalences being higher in the rare disease sample compared to normative population data. Regarding *emotional food avoidance*, a higher prevalence was found in the normative population data compared to the rare disease sample. Effect sizes ranged from poor to moderate. See Supplementary Table 1 for more information on the prevalence of symptoms of ARFID at the item level.

#### Self-report and parent report data

Self-report and parent report symptoms of ARFID, eating disorder psychopathology, HRQoL, and mental health are shown in Table 3. For parent report data, no differences were found for symptoms of ARFID between mothers and fathers, *t*(333.03) = 0.52, *p* $\:=$ 0.592, *d* $\:=$ 0.05. Nevertheless, mothers did not report lower child HRQoL scores, *t*(372.85) = 2.41, *p* = 0.092, *d* = -0.17, but reported higher child mental health scores than fathers, *t*(337.80) = -1.69, *p* = 0.016, *d* = 0.23.

**Table 3:** Means, standard deviations of EDY-Q, ChEDE-Q8, DCGM-37, and YSR/11-18R and CBCL/6-18R subscales

Scale	Self-reported		Parent reported	
	*n*	M (SD)	*n*	M (SD)
EDY-Q/ PEDY-Q total score	153	2.2 (0.77)	459	2.3 (0.94)
ChEDE-Q8 global score	159	1.6 (1.12)	-	-
DCGM-37 total score	161	68.6 (17.75)	477	62.6 (18.45)
YSR/11-18R & CBCL/6-18R total score	158	37.1 (23.47)	473	32.5 (24.76)

*Note. M =* Mean, *SD =* Standard deviation. EDY-Q = Eating Disorders in Youth – Questionnaire. PEDY-Q = Parent Eating Disorders in Youth – Questionnaire. ChEDE-Q8 = Eating Disorder Examination-Questionnaire 8 for Children. DCGM-37 = DISABKIDS Chronic Generic Modul-37. YSR/11-18R = Youth Self-Report/11-18R. CBCL/6-18R = Child Behavior Checklist/6-18R

**Fig. 1:**
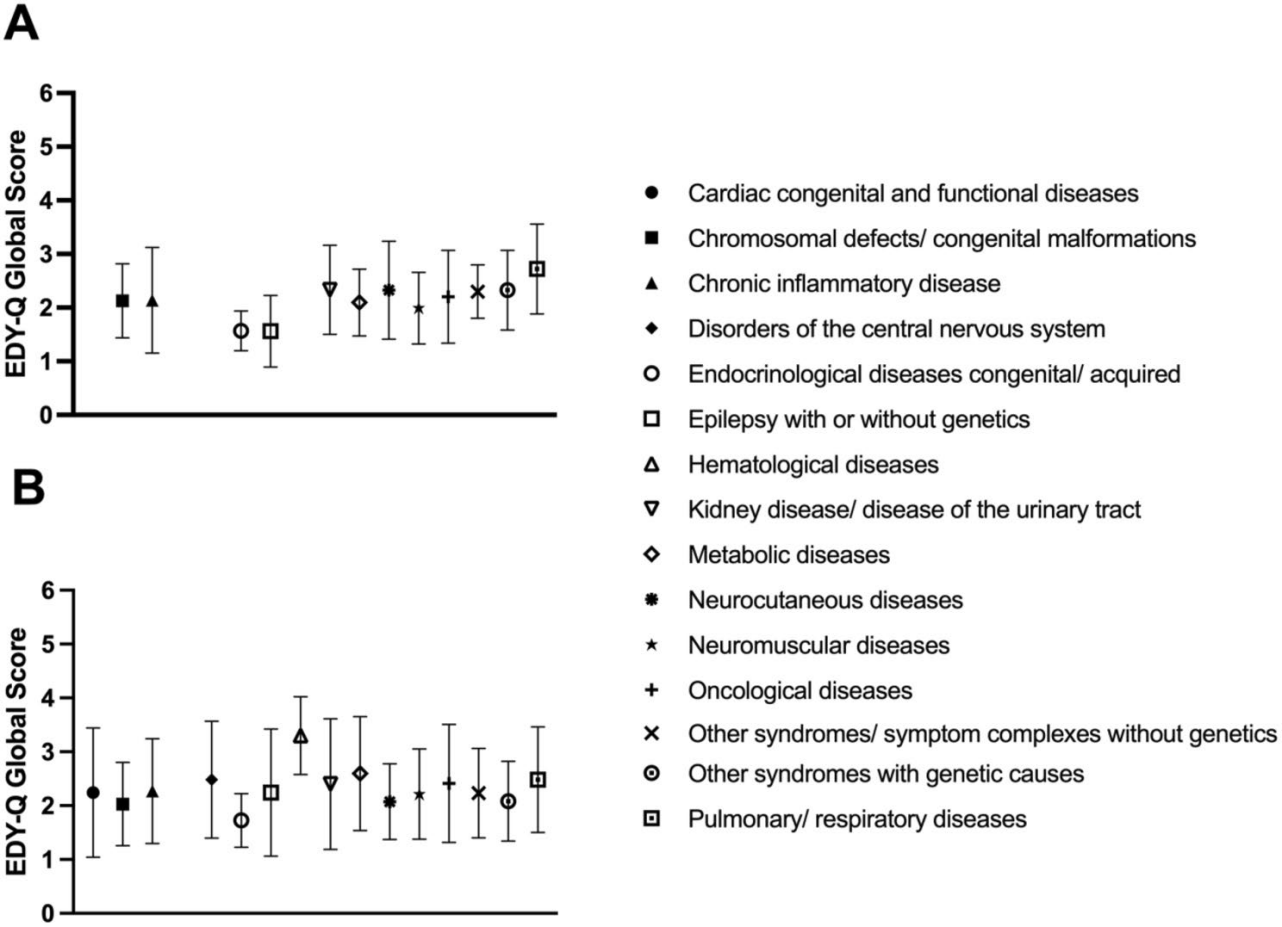
Differences in symptoms of ARFID between rare disease groups, as reported in self-report (**A**) and parent report (**B**). Note. EDY-Q and PEDY-Q total score is given in means $\:\pm\:$ standard deviations. (**A**) represents self-reported symptoms of ARFID. (**B**) represents parent reported symptoms of ARFID. Due to the very small sample size of some rare disease groups, these have been excluded from the figure; however, they are briefly listed below. Self-report: Cardiac congenital and functional diseases, Chronic intestinal diseases, Hematological diseases; Parent report: Chronic intestinal diseases

**Fig. 2:**
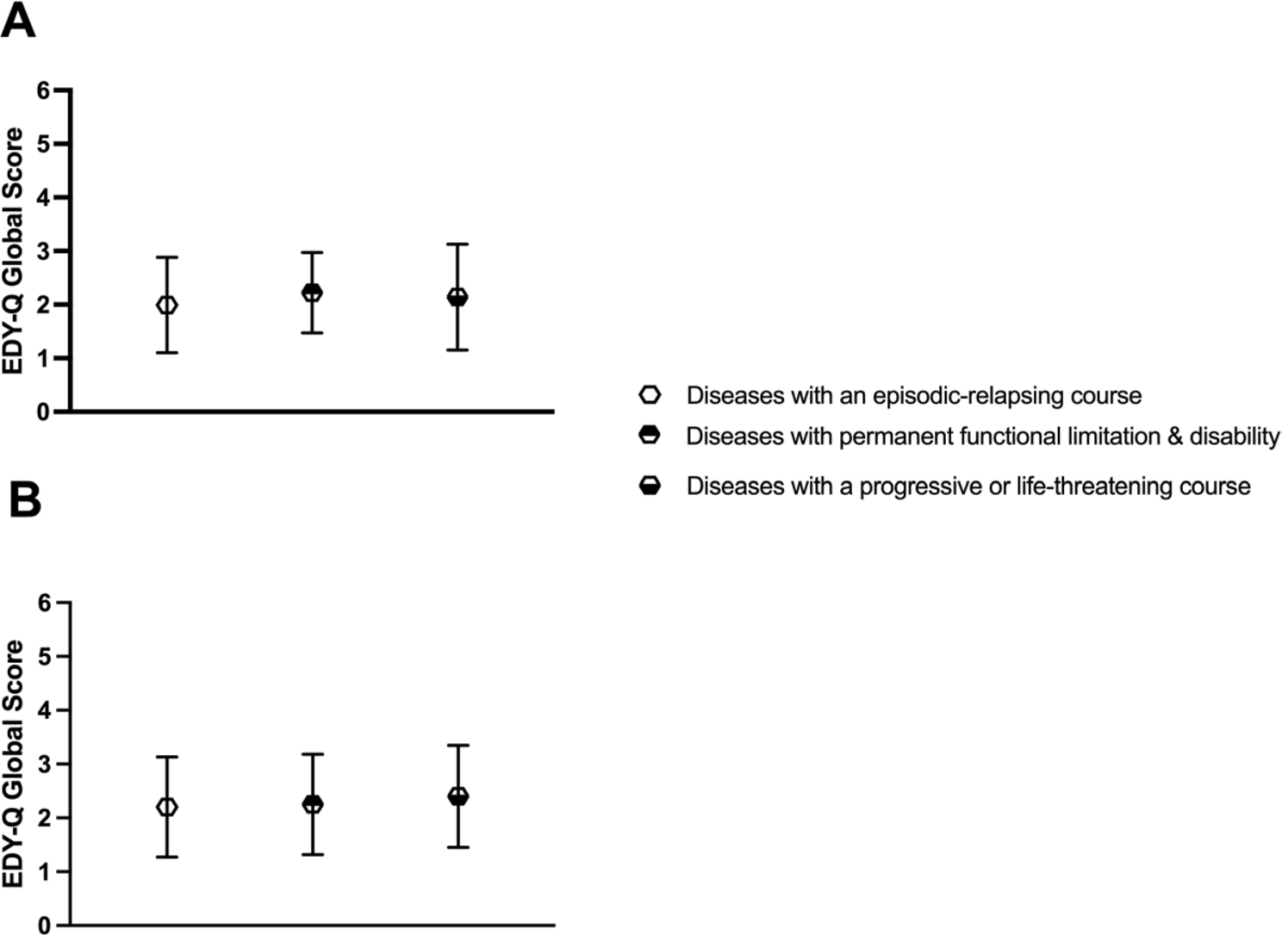
Differences in symptoms of ARFID between superordinate groups, accounting for disease severity, as observed in self-report (**A**) and parent report (**B**). Note. EDY-Q and PEDY-Q total score is given in means standard deviations. (**A**) represents self-reported symptoms of ARFID. (**B**) represents parent reported symptoms of ARFID

No difference between subgroups of rare diseases in the total score of self-reported symptoms of ARFID was found, *H* [15] = 17.00, *p* = 0.319, $\:{{\upeta\:}}^{2}$ = 0.01. In contrast, subgroup analyses among rare disease groups revealed relevant differences in the total score of parent reported symptoms of ARFID, *H* [15] = 29.47, *p* = 0.014, $\:{{\upeta\:}}^{2}$ = 0.02. The rare disease groups with the highest scores on symptoms of ARFID include chronic inflammatory diseases, pulmonary/respiratory diseases, and metabolic diseases. Moreover, there were no differences between the groups in the total score of self-reported symptoms of ARFID, *H* [2] = 3.679, *p* = 0.262, $\:{{\upeta\:}}^{2}$ < 0.01, and in the total score of parent reported symptoms of ARFID, *H* [2] = 3.545, *p* = 0.179, $\:{{\upeta\:}}^{2}$ = 0.01, when considering superordinate groups that account for the severity of the diseases. See Figs. 1 and 2 for differences in symptoms of ARFID between rare disease groups.

#### Children and adolescents with and without symptoms of ARFID

Children and adolescents with symptoms of ARFID based on self-report had higher prevalence in a third of the items compared to those without symptoms of ARFID. Effect sizes ranged from poor to moderate. In contrast, no relevant difference between groups was found for eating disorder psychopathology. Children and adolescents with and without symptoms of ARFID, based on parent report, differed regarding all items but *emotional food avoidance.* Effect sizes ranged from poor to large. See Supplementary Table 2 for more information regarding children and adolescents with and without symptoms of ARFID on item level. Moreover, no difference emerged between children and adolescents with and without symptoms of ARFID regarding self-reported HRQoL, *U* = 570.5, *p* = 0.346, *g* = 0.28, and mental health scores, *U* = 560.0, *p* = 0.508, *g* = 0.25. No relevant difference was found between children and adolescents with and without symptoms of ARFID regarding parent reported HRQoL, *U* = 10272.0, *g* = 0.06, *p* = 0.662, and mental health scores, *U* = 11555.5, *p* = 0.389, *g* = 0.21. Additionally, the descriptive characteristics of children and adolescents with symptoms of ARFID are given in Supplementary Tables 3 to 5.

### Correlational analyses

Table 4 shows the bivariate association of self-reported and parent reported variables. No relevant association was found between symptoms of ARFID and age or gender. Nevertheless, symptoms of ARFID were negatively associated with HRQoL and mental health in both self-reported and parent reported data, with more symptoms of ARFID corresponding to more impairment in HRQoL and mental health. Pearson correlations ranged from small to large.

**Table 4:** Correlation of self-reported and parent reported data of the EDY-Q, DCGM-37, YSR/11-18R & CBCL/6-18R, and self-reported ChEDE-Q8

Variables	1	2	3	4	5
Self-Report (*n* = 169)	Parent report (*n* = 502)				
1. Age	-	0.07 (0.097)	− 0.04 (0.408)	**− 0.20 (< 0.001)**	− 0.05 (0.261)
2. Sex	0.08 (0.333)	-	0.03 (0.531)	0.01 (0.786)	− 0.01 (0.908)
3. EDY-Q/ PEDY-Q total score	− 0.02 (0.839)	0.05 (0.502)	-	**− 0.13 (0.006)**	**0.20 (< 0.001)**
4. DCGM-37 total score	**− 0.18 (0.022)**	− 0.04 (0.616)	**− 0.25 (0.002)**	-	**0.58 (< 0.001)**
5. YSR/11-18R & CBCL/6-18R total score	0.11 (0.153)	0.09 (0.272)	**0.28 (< 0.001)**	**− 0.53 (< 0.001)**	-
6. ChEDE-Q8 global score*	**0.16 (0.038)**	**0.195 (0.013)**	0.132 (0.102)	**− 0.29 (< 0.001)**	**0.46 (< 0.001)**

Note. Main entries are Pearson *r*, with *p* values in parenthesis. Biological sex was used for this analysis to generate a binary variable. Sex: 0 = male and 1 = female. EDY-Q = Eating Disorders in Youth – Questionnaire. PEDY-Q = (Parent) Eating Disorders in Youth – Questionnaire. DCGM-37 = DISABKIDS Chronic Generic Modul-37. YSR/11-18R = Youth Self-Report/11-18R. CBCL/6-18R = Child Behavior Checklist/6-18R. ChEDE-Q8 = Eating Disorder Examination-Questionnaire 8 for Children. * Only the self-report was available

## Discussion

Previous research on ARFID has focused on general population and pediatric population samples, as well as specialized treatment services [5, 6, 28, 36]. To our current knowledge, this is the first study to investigate ARFID in a large clinical sample of 8- to 21-year-old children and adolescents with rare diseases via self-report and parent report data.

In this study, a relatively high prevalence of symptoms of ARFID in both self-reports and especially parent reports were found. The prevalence of symptoms of ARFID were higher in the rare disease sample compared to normative population data [28] and clinical samples [6, 37]. These findings were also evident when using different diagnostic codings of the instrument EDY-Q [26–28], with prevalences ranging from 3.6% for symptoms of ARFID with underweight problems to 34.9% for broad symptoms of ARFID without underweight problems in the self-report, and from 10.2% for symptoms of ARFID with underweight problems to 35.3% for broad symptoms of ARFID without underweight problems in the parent report. Moreover, the prevalence of ARFID symptoms differed between self-reports and parental reports in cases where both types of information were available, with parental reports indicating a higher prevalence than self-reports, exhibiting small to moderate effect sizes. Possible explanations for this divergence may include that parents have different points of comparison, such as siblings or other family members. While some symptoms of ARFID are easily observable by parents, others, such as specific eating-related fears, may be less noticeable. Additionally, parents and children might interpret behaviors differently; behavior that a child considers normal or unproblematic might be perceived by a parent as a warning sign. Furthermore, differences in cognitive development between children and parents in perceiving and verbalizing symptoms may lead to diagnostic disagreements [37].

The findings on ARFID symptoms were evident when comparing item-level symptoms between self-report data and normative population data [28], with a higher prevalence observed in the self-report data for half of the items. A moderate effect was only found for the item *selective eating behavior*, while small effects were found for the items *wish to gain weight*, *shape concern*, and *sensory food avoidance*. The finding that the cohort of children and adolescents with a rare disease have clinically relevant more *selective eating behavior* compared to the normative population data is in line with previous research that established a link between dysfunctional eating behaviors and neurodevelopmental (e.g., autism) and somatic (e.g., respiratory) conditions [6, 9, 38]. Nevertheless, the small effects found for the items *wish to gain weight* and *shape concern* in the rare diseases sample compared to the normative population sample might be due to the fact that these symptoms are more common with older age in children and adolescents as well as in girls [39], since the rare disease sample was comparatively older than the normative population sample.

Regarding specific rare disease groups, differences in the overall score of symptoms of ARFID between groups were found in the parent reported version but not in the self-report version. Nevertheless, a close examination of children and adolescents with symptoms of ARFID showed that certain groups, such as pulmonary/respiratory diseases, metabolic diseases, and neuromuscular diseases, were affected more frequently [40]. One possible explanation for the higher prevalence of ARFID symptoms in these groups may be the complex interplay of medical complications, unique nutritional needs, frequent medical interventions, and heightened sensory sensitivities [13–16]. For example, children and adolescents with metabolic diseases often require strict dietary restrictions and specialized nutrition, which may lead to anxiety around food and contribute to the development of ARFID symptoms [41]. The absence of relevant differences in the overall ARFID symptoms score between superordinate groups [23] in both the self-report and parent report versions suggests that symptoms of ARFID may be consistent across varying severity levels of coexisting rare diseases. Therefore, it is important to recognize that ARFID symptoms can manifest independently of the severity of these diseases, despite the variability in how the individual conditions express themselves.

Additionally, this study replicated and expanded upon previous research [6, 27, 42] by demonstrating that children and adolescents with symptoms of ARFID, compared to those without symptoms of ARFID, self-reported more *underweight problems*, *wish to gain weight*, and *fear of swallowing* with a moderate effect, and *sensory food avoidance* with a small effect. In contrast, in children and adolescents with versus without symptoms of ARFID, parents reported more *underweight* with a large effect and more *wish to gain weight* with a moderate effect. Moreover, other symptoms of ARFID that were more prevalent in children and adolescents with symptoms of ARFID compared to those without symptoms, with a small effect included *food avoidance*, *lack of interest in food*, *selective eating behavior*, *fear of choking*, *fear of swallowing*, and *sensory food avoidance*. Furthermore, children and adolescents with rare diseases and with versus without symptoms of ARFID did not differ regarding self-reported eating disorder psychopathology. In summary, the self- and parent reported clinically relevant results on children and adolescents with symptoms of ARFID, compared to those without symptoms of ARFID, along with the not relevant difference in self-reported eating disorder psychopathology are consistent with previous research, suggesting that eating behavior is not motivated by body image concerns [6, 42, 43].

Furthermore, the results showed that both self-reported and parent reported ARFID symptoms were associated with impairments in HRQoL and mental health in children and adolescents with rare diseases but were not associated with age or gender. While the association of ARFID with impairment in HRQoL and mental health is consistent with previous research, the lack of association with demographics contrasts with prior studies [44]. One possible explanation for this lack of association between ARFID symptoms and demographics like age and gender in this study could be the unique characteristics of the rare disease population, which may overshadow typical demographic influences.

### Strengths and limitations

The present study has notable strengths, including the large sample size of children and adolescents with different rare diseases and the assessment of both self-reported and parent reported symptoms of ARFID. Nonetheless, some constraints must be taken into account when evaluating the results. The heterogeneity of the rare diseases included in this study ranged from an episodic-relapsing course to a progressive or life-threatening course, complicating the interpretation of results across different disease groups. While some groups of rare diseases were well represented, this was not the case for all groups; therefore, the results for superordinate groups cannot necessarily be extrapolated. Additionally, some ARFID categories at the category and item levels were fulfilled by a rather small number of persons, which may limit the ability to make meaningful comparisons with the rest of the sample. Moreover, there is substantial variability in the expression of the disease groups as well as their associated nutritional needs. Different nutritional requirements, such as specialized diets, hypercaloric diets, or tube feeding, may influence the symptoms of ARFID and complicate comparisons among different rare disease groups. This variability thus decreases the generalizability of the results. Furthermore, the study only included data from children and adolescents aged eight years and older. Previous research has identified ARFID symptoms occurring earlier in life [45]. Moreover, a limitation is that the analyses did not include medical record data of participants and interview-based assessment [43, 46]; therefore, a diagnosis of ARFID could not be determined [47, 48]. Moreover, comorbidities with psychiatric and neurodevelopmental disorders are not included in the results and therefore may limit the understanding of the full clinical profile of the participants.

## Conclusion

This study documented a relatively high prevalence of symptoms of ARFID in children and adolescents with rare diseases, emphasizing the clinical relevance of this eating disorder in this cohort. Even though rare diseases and ARFID present a double risk—partly because healthcare professionals often lack knowledge of both—their comorbidities should be carefully considered in clinical care and research [49, 50]. For this purpose, screening instruments should be developed alongside methods such as interview-based assessments and nutritional evaluations in the clinical care of rare diseases. This type of screening may facilitate the timely implementation of targeted behavioral interventions, cognitive-behavioral therapy, family therapy, or a synergistic approach that integrates these modalities [51]. Since some groups of rare diseases may phenomenologically present a particular risk for symptoms of ARFID, future research should further investigate the relationship between specific rare disease groups and ARFID.

## Supplementary Information

Below is the link to the electronic supplementary material.

Supplementary Material 1

## Data Availability

The datasets generated during the current study are available from the corresponding author on reasonable request.

## Abbreviations

ARFID: Avoidant/restrictive food intake disorder
ChEDE-Q8: Eating Disorder Examination-Questionnaire 8 for Children
CBCL/6-18R: Child Behavior Checklist/6-18R
EDY-Q: Eating Disorders in Youth-Questionnaire
DCGM-37: DISABKIDS Chronic Generic Modul-37
HRQoL: Health-related quality of life
ICC: Intraclass correlation coefficients
YSR/11-18R: Youth Self-Report/11-18R
