# Supplementary material for: Prevalence of avoidant/restrictive food intake disorder in children and adolescents with rare diseases
Source: Orphanet J Rare Dis. 2026 Jan 31;21:85. doi: 10.1186/s13023-026-04233-5 (PMC12958663; doi:10.1186/s13023-026-04233-5)
Supplement: Supplementary file 1 — Supplementary Material 1 [file 13023_2026_4233_MOESM1_ESM.docx]

**Supplementary Table 1.** Prevalence of self-reported symptoms of ARFID for the rare diseased sample and norm data based on the Eating Disorders in Youth‐Questionnaire.

|  | **Rare disease sample (*n* = 169)** | **Normative data (*n* = 799) ^a^** | **Fisher’s exact test** | |
| --- | --- | --- | --- | --- |
|  | *n* (%) | *n* (%) | *p* | *V* |
| EDY-Q items reported at least ‘often’ ($\geq4)$ |  |  |  |  |
| 1. Food avoidance | 8/163 (4.7%) | 47 (5.9%) | .764 | .02 |
| 2. Lack of interest in food^a^ | 12/164 (7.1%) | 96 (12.0%) | .110 | .06 |
| 3. Emotional food avoidance | 30/163 (17.8%) | 228 (28.5%) | **.010** | .09 |
| 4. Underweight^a^ | 32/164 (18.9%) | 93 (11.6%) | **.009** | .09 |
| 5. Wish to gain weight | 24/163 (14.2%) | 58 (7.3%) | **.003** | .10 |
| 6. Weight concern (feelings of fatness)^a^ | 36/162 (22.2%) | 148 (18.5%) | .33 | .04 |
| 7. Shape concern^a^ | 58/159 (36.5%) | 135 (16.9%) | **<.001** | .18 |
| 8. Selective eating behavior | 91/162 (53.8%) | 293 (36.7%) | **<.001** | .31 |
| 9. Avoidance to try new foods | 73/162 (43.2%) | 220 (27.5%) | **<.001** | .14 |
| 10. Fear of choking^a^ | 4/163 (2.4%) | 36 (4.5%) | .327 | .04 |
| 11. Fear of swallowing | 1/163 (0.6%) | 14 (1.8%) | .471 | .03 |
| 12. Sensory food avoidance^a^ | 58/164 (34.3%) | 121 (15.1%) | **<.001** | .20 |
| Note. ARFID = Avoidant/Restrictive Food Intake Disorder. EDY-Q = Eating Disorders in Youth – Questionnaire. ^a^ Used as indicators to assess the manifestation of symptoms in ARFID. ^a^ Normative data were derived from Schmidt, R., Vogel, M., Hiemisch, A., Kiess, W., & Hilbert, A. (2018). Pathological and non-pathological variants of restrictive eating behaviors in middle childhood: A latent class analysis. *Appetite*, *127*, 257–265. | | | | |

**Supplementary Table 2.** Group differences in clinical variables between children and adolescents with and without symptoms of ARFID based on self‐report (*n* = 169) and parent report (*n* = 502) data of the Eating Disorders in Youth‐Questionnaire.

|  | **Self-report** | | **Parent report** | | **Fisher’s exact test self-report** | | **Fisher’s exact test**  **parent report** | |  |
| --- | --- | --- | --- | --- | --- | --- | --- | --- | --- |
|  | Yes (*n* = 6) | No (*n* = 163) | Yes (*n* = 51) | No (*n* = 451) |  |  |  |  |  |
|  | *n* (%) exceeding cut-off | *n* (%) exceeding cut-off | *n* (%) exceeding cut-off | *n* (%) exceeding cut-off | *p* | *V* | *p* | *V* |  |
| 1. Food avoidance | 1/6 (16.7%) | 7/157 (4.5%) | 20/50 (40.0%) | 42/435 (9.7%) | .264 | .11 | **<.001** | .28 |  |
| 2. Lack of interest in food^a^ | 1/6 (16.7%) | 11/158 (7.0%) | 21/51 (41.2%) | 52/436 (11.9%) | .371 | .07 | **<.001** | .25 |  |
| 3. Emotional food avoidance | 1/6 (16.7%) | 29/157 (18.5%) | 10/49 (20.4%) | 74/433 (17.1%) | .999 | .01 | .553 | .03 |  |
| 4. Underweight^a^ | 6/6 (100.0%) | 26/158 (16.5%) | 51/51 (100.0%) | 59/438 (13.5%) | **<.001** | .40 | **<.001** | .63 |  |
| 5. Wish to gain weight | 4/5 (80.0%) | 20/158 (12.7%) | 23/48 (47.9%) | 31/429 (7.2%) | **.002** | .33 | **<.001** | .39 |  |
| 6. Weight concern (feelings of fatness)^a^ | 0/6 (0.0%) | 36/156 (23.1%) | 0/51 (0.0%) | 95/428 (22.2%) | .403 | .11 | **<.001** | .17 |  |
| 7. Shape concern^a^ | 0/6 (0.0%) | 58/153 (37.9%) | 0/51 (0.0%) | 174/411 (42.3%) | .087 | .15 | **<.001** | .27 |  |
| 8. Selective eating behavior | 3/6 (50.0%) | 88/156 (56.4%) | 36/50 (72.0%) | 232/435 (53.3%) | .999 | .02 | **.016** | .11 |  |
| 9. Avoidance to try new foods | 4/6 (66.7%) | 69/156 (44.2%) | 34/51 (66.7%) | 212/435 (48.7%) | .410 | .09 | **.018** | .11 |  |
| 10. Fear of choking^a^ | 1/6 (16.7%) | 3/157 (1.9%) | 12/51 (23.5%) | 21/434 (4.8%) | 141 | .18 | **<.001** | .23 |  |
| 11. Fear of swallowing | 1/6 (16.7%) | 0/157 (0.0%) | 13/51 (25.5%) | 15/432 (3.5%) | **<.001** | .40 | **<.001** | .29 |  |
| 12. Sensory food avoidance^a^ | 6/6 (100.0%) | 52/158 (32.9%) | 37/51 (72.5%) | 151/435 (34.7%) | **.002** | .26 | **<.001** | .24 |  |
| ChEDE-Q8 global score^b^ | 1/6 (16.7%) | 17/139 (10.9%) | - | - | .513 | .04 | - | - |  |
| Note. ARFID = Avoidant/Restrictive Food Intake Disorder. ChEDE-Q8: Eating Disorder Examination-Questionnaire 8 for Children. EDY-Q = Eating Disorders in Youth – Questionnaire. ^a^ Used as indicators to assess the manifestation of symptoms in ARFID. *n* (%) exceeding cut-off was defined as EDY-Q items reported at least ‘often’ ($\geq4)$. ^b^ The ChEDE-Q8 scores exceeded the cut-off when they were in the ≥90th percentile. | | | | | | | | | |

**Supplementary Table 3.** Descriptive information on children and adolescents with ARFID symptoms based on self-report data.

| **No.** | **Sex** | **Age (in years)** | **Rare disease group** | **Medical diagnosis** | **ChEDE-Q8 global score** |
| --- | --- | --- | --- | --- | --- |
| 1 | f | 10 | Kidney disease/ disease of the urinary tract | Unspecified | 1.25 |
| 2 | f | 11 | Kidney disease/ disease of the urinary tract | Renal hypoplasia | 1.00 |
| 3 | m | 12 | Neuromuscular diseases | Muscular dystrophy | 2.25 |
| 4 | m | 15 | Neuromuscular diseases | Centronuclear myopathy | 1.50 |
| 5 | m | 19 | Neuromuscular diseases | Duchenne muscular dystrophy | 1.63 |
| 6 | f | 19 | Pulmonary / respiratory diseases | Cystic fibrosis | 3.25 |
| Note. ARFID = Avoidant/Restrictive Food Intake Disorder. EDY-Q = Eating Disorders in Youth-Questionnaire. ChEDE-Q8 = Eating Disorder Examination-Questionnaire 8 for Children. | | | | | |

**Supplementary Table 4.** Descriptive information on female children and adolescents with ARFID symptoms based on parent report data.

| **No.** | **Sex** | **Age (in years)** | **Rare disease group** | **Medical diagnosis** |
| --- | --- | --- | --- | --- |
| 1 | f | 8 | Metabolic diseases^a^ | Alexander disease |
| 2 | f | 8 | Metabolic diseases^a^ | Alexander disease |
| 3 | f | 8 | Metabolic diseases | Unspecified |
| 4 | f | 8 | Metabolic diseases | Medium chain acyl-CoA dehydrogenase deficiency |
| 5 | f | 8 | Metabolic diseases | D-2-hydroxyglutaric aciduria |
| 6 | f | 8 | Chronic inflammatory disease | Autoimmune encephalitis |
| 7 | f | 9 | Epilepsy with or without genetics^a^ | 9q21.13 microdeletion syndrome |
| 8 | f | 9 | Epilepsy with or without genetics^a^ | 9q21.13 microdeletion syndrome |
| 9 | f | 10 | Other syndromes with genetic causes | Turner syndrome |
| 10 | f | 10 | Neuromuscular diseases^a^ | Spinal muscular atrophy type 2 |
| 11 | f | 10 | Neuromuscular diseases^a^ | Spinal muscular atrophy type 2 |
| 12 | f | 11 | Other syndromes/ symptom complexes without genetics | Unspecified |
| 13 | f | 11 | Kidney disease / disease of the urinary tract | Renal hypoplasia |
| 14 | f | 12 | Chromosomal defects/ congenital malformations | Corpus callosum agenesis |
| 15 | f | 12 | Epilepsy with or without genetics^a^ | Unspecified |
| 16 | f | 12 | Epilepsy with or without genetics^a^ | Unspecified |
| 17 | f | 13 | Disorders of the central nervous system | Congenital portosystemic shunt |
| 18 | f | 13 | Neuromuscular diseases^a^ | Spinal Muscular Atrophy Type 2 |
| 19 | f | 13 | Neuromuscular diseases^a^ | Spinal Muscular Atrophy Type 2 |
| 20 | f | 15 | Other syndromes with genetic causes | Smith-Magenis syndrome |
| 21 | f | 18 | Chronic inflammatory disease | Pediatric multiple sclerosis |
| 22 | f | 18 | Metabolic diseases^a^ | Homocystinuria |
| 23 | f | 18 | Metabolic diseases^a^ | Homocystinuria |
| Note. ARFID = Avoidant/Restrictive Food Intake Disorder. EDY-Q = Eating Disorders in Youth-Questionnaire. ChEDE-Q8 = Eating Disorder Examination-Questionnaire 8 for Children. ^a^ Cases where both mothers and fathers reported that the child exhibited symptoms of ARFID. | | | | |

**Supplementary Table 5.** Descriptive information on male children and adolescents with ARFID symptoms based on parent report data.

| **No.** | **Sex** | **Age (in years)** | **Rare disease group** | **Medical diagnosis** |
| --- | --- | --- | --- | --- |
| 1 | m | 8 | Neuromuscular diseases | Unspecified |
| 2 | m | 9 | Neuromuscular diseases^a^ | Duchenne muscular dystrophy |
| 3 | m | 9 | Neuromuscular diseases^a^ | Duchenne muscular dystrophy |
| 4 | m | 9 | Neuromuscular diseases^a^ | Duchenne muscular dystrophy |
| 5 | m | 9 | Neuromuscular diseases^a^ | Duchenne muscular dystrophy |
| 6 | m | 9 | Hematological diseases | Autoimmune Neutropenia |
| 7 | m | 9 | Other syndromes with genetic causes | Silver-Russell syndrome |
| 8 | m | 10 | Other syndromes/ symptom complexes without genetics | Unspecified |
| 9 | m | 11 | Epilepsy with or without genetics | Unspecified |
| 10 | m | 11 | Pulmonary/ respiratory diseases | Mucopolysaccharidosis type 4 |
| 11 | m | 11 | Kidney disease / disease of the urinary tract | Unspecified |
| 12 | m | 11 | Cardiac congenital and functional diseases | Hypoplastic left heart syndrome |
| 13 | m | 12 | Other syndromes with genetic causes | Cystic Fibrosis |
| 14 | m | 12 | Metabolic diseases | Phenylketonuria |
| 15 | m | 12 | Chronic intestinal diseases | Eosinophilic Esophagitis |
| 16 | m | 13 | Chronic inflammatory disease | Muckle-Wells syndrome |
| 17 | m | 15 | Neuromuscular diseases^a^ | Centronuclear myopathy |
| 18 | m | 15 | Neuromuscular diseases^a^ | Centronuclear myopathy |
| 19 | m | 15 | Metabolic diseases | Phenylketonuria |
| 20 | m | 15 | Epilepsy with or without genetics | Juvenile absence epilepsy |
| 21 | m | 15 | Metabolic diseases | Lysinuric protein intolerance |
| 22 | m | 15 | Metabolic diseases | GM1 gangliosidosis |
| 23 | m | 15 | Other syndromes with genetic causes | Cystic Fibrosis |
| 24 | m | 16 | Neuromuscular diseases | Myotonic dystrophy type 1 |
| 25 | m | 16 | Epilepsy with or without genetics | Unspecified |
| 26 | m | 18 | Neurocutaneous diseases | Neurofibromatosis type 1 |
| 27 | m | 19 | Neuromuscular diseases | Duchenne muscular dystrophy |
| 28 | m | 19 | Oncological diseases | Glioblastoma |
| Note. ARFID = Avoidant/Restrictive Food Intake Disorder. EDY-Q = Eating Disorders in Youth-Questionnaire. ChEDE-Q8 = Eating Disorder Examination-Questionnaire 8 for Children. ^a^ Cases where both mothers and fathers reported that the child exhibited symptoms of ARFID. | | | | |
